# Effect of Deficit Irrigation on Agronomic and Physiological Performance of Pomegranate (*Punica granatum* L.)

**DOI:** 10.3390/plants14020164

**Published:** 2025-01-08

**Authors:** Rossana Porras-Jorge, José Mariano Aguilar, Carlos Baixauli, Julián Bartual, Bernardo Pascual, Nuria Pascual-Seva

**Affiliations:** 1 Departamento de Producción Vegetal, Universitat Politècnica de València, Camí de Vera, s/n, 46022 Valencia, Spain; zrporras@doctor.upv.es; 2 Centro Experiencias Cajamar Paiporta, C. Cementerio Nuevo s/n, 46200 Paiporta, Spain; josemarianoaguilar@fundacioncajamar.com (J.M.A.); carlosbaixauli@fundacioncajamar.com (C.B.); 3 Estación Experimental Agraria de Elche (SST), CV-855, Km. 1, 03290 Elche, Spain; bartual_jul@gva.es; 4 Centro Valenciano de Estudios sobre el Riego, Universitat Politècnica de València, Camí de Vera, s/n, 46022 Valencia, Spain; bpascual@upv.es

**Keywords:** ‘Mollar de Elche’, sustained deficit irrigation, regulated deficit irrigation, water productivity

## Abstract

Agriculture accounts for over 70% of global freshwater consumption, with increasing competition for water resources due to climate change and rising urban and industrial demands. This study analyzes the effect of deficit irrigation (DI) on the agronomic and physiological performance of pomegranate (*Punica granatum* L.) in a Mediterranean climate. Deficit irrigation strategies, including sustained deficit irrigation (SDI) and regulated deficit irrigation (RDI), were evaluated against a control with full irrigation. The research was conducted over two growing seasons (2022–2023) at the Cajamar Experimental Centre in Paiporta, Valencia, Spain. RDI strategies achieved approximately 10% water savings without compromising marketable yield or fruit weight, while SDI resulted in significant water savings (~50%) but with a notable reduction in marketable yield, particularly in hot and dry conditions. SDI also reduced tree growth in height and trunk diameter compared to RDI and control strategies. The study concludes that RDI is a viable irrigation strategy for pomegranate cultivation under water-limited conditions, whereas SDI should be reserved for situations of severe water scarcity.

## 1. Introduction

Agriculture consumes over 70% of the planet’s freshwater [1]. In recent years, freshwater scarcity has become a significant problem, especially in arid areas, increasing the competition for water among agricultural, industrial, and urban consumers. Rapid population growth, different human activities, and the greater incidence of droughts caused by climate change are factors that increase this problem. As urban and industrial demands for water grow, agriculture will be pressured to make more efficient use of its shrinking allocation. The Mediterranean climate is characterized by mild winter temperatures and long, hot, and dry summers, with rainfall subject to high interannual and seasonal variability, making irrigation essential for crop production. The widespread water limitations for agriculture require developing strategies to make the most of irrigation water.

Deficit irrigation (DI) is an irrigation practice through which crops are irrigated with lower amounts of water than those that plants need for optimal growth and development [2,3,4]. It includes continuous or sustained deficit irrigation (SDI) and controlled or regulated deficit irrigation (RDI) [5]. SDI imposes water deficit uniformly throughout the crop cycle, thus avoiding significant water stress in any phenological phase that could significantly affect marketable yield. RDI is deficit irrigation based on phases or stages, which consists of imposing water deficits in specific phenological phases in which crops are less sensitive to water stress [5].

The pomegranate tree (*Punica granatum* L.) is one of the oldest known fruit trees; its origin extends from the Balkans to the Himalayas [6]. Pomegranate cultivation began in prehistoric times, and its domestication probably began in the Neolithic era in the Transcaucasian–Caspian region and northern Turkey [7]. The Phoenicians, Greeks, and Egyptians cultivated it. The Phoenicians spread its cultivation along the shores of the Mediterranean [8]. They cultivated it around Carthage, which is why the Romans named this fruit *Malum punicatum* (the Carthaginian apple). Later, due to the attractive appearance of the arils (seeds) packed in the carpels [9], Columella gave it the name *Malum granatum*, or grain apple, from which comes the names pomegranate in English, grenade in French, granada in Spanish, and melagrana in Italian. Due to the many seeds it contains, the pomegranate was considered as an emblem of fertility in ancient times, being mentioned in the Bible (Numbers 13:23; Deuteronomy 8:8; Exodus 28:33–35; 1 Kings 7:18–20; Songs 4:3, 4:13, 8:2) [10].

The pomegranate is a fruit-bearing deciduous tree that can adapt to different climatic conditions, as demonstrated by its wide distribution in the wild form throughout Eurasia to the Himalayas. It is perfectly acclimated to the Mediterranean edaphoclimatic conditions, being considered a crop tolerant of soil water deficit [7,11]. Nevertheless, to reach optimal yield and fruit quality and reduce the incidence of fruit physiological disorders, traditionally, pomegranate trees have been regularly irrigated throughout the season. Although it is considered a minor fruit tree (also known as a neglected and underutilized species) [12], it is acquiring great interest both in Spain and worldwide, being considered an emerging fruit crop [13] for its organoleptic characteristics and its beneficial effects on human health due to its high content of antioxidants [6,11,14]. Thus, it is an interesting option for diversifying fruit production in the Mediterranean basin.

Rodriguez et al. [15] stated that ‘Mollar de Elche’ (‘ME’) pomegranate trees face water stress by developing stress avoidance and tolerance mechanisms. From the beginning of the stress period, leaf conductance decreases to control water loss and avoid stress. Near the end of the stress period, maximum stress levels are developed, and an active osmotic adjustment is triggered, favoring the maintenance of leaf turgor (stress tolerance mechanism).

The interest that deficit irrigation has raised in pomegranate cultivation in semi-arid climates is demonstrated in the research works that have been carried out on this subject in the province of Alicante (southeast Spain) on ‘ME’. In two companion papers [11,16], authors tested both SDI and RDI in different periods, concluding that irrigation restriction during the flowering and fruit set could be used in water scarcity situations; however, it led to small water savings (9–14%). The flowering period was identified as a non-critical period for RDI application [14], as it generated a 5.25% lower commercial yield, with a water saving of 24%. Pomegranate fruits are sensitive to water restrictions applied during the final phases of fruit growth and ripening [17,18]; the authors stated that fruit ripening is a critical period, and thus, irrigation is essential in this phenological period to achieve maximum yield. When SDI was tested [11,16], it produced more but lighter fruits. Galindo et al. [13] reviewed the state of the art regarding DI and the response of four emerging fruit crops, including pomegranate. In the review, the authors collected the studies by the leading research teams in DI of the pomegranate tree. Volschenk [19] reviewed in detail research conducted on pomegranate trees regarding irrigation methods, crop water requirements, water use efficiency and productivity, and water management strategies under conditions of limited water supply. She subsequently carried out another review [20] focused mainly on the effects of water deficits on physiology, vegetative and fruit growth, and yield and fruit quality of the pomegranate. She concluded that the results of one study could not simply be transferred to another area where conditions may be different given the variable results obtained with different systems and cultivars in different countries, which require conducting research under local conditions.

Given that the response of pomegranate trees to water restrictions differs depending on the different phenological phases in which they are applied and that the published results on water savings and the yields obtained with DI are not always coincident, particularly in the initial stages of the growing season, the objective of this research was to evaluate the agronomic behaviour of seven-year-old pomegranate trees under an SDI and two RDI strategies in the initial stages [one during the flowering–setting phase (RDI1) and another during the open flower–early fruit growth phase with color change (RDI2), which, to the authors’ knowledge, has not been studied] and to establish a schedule of irrigation for growers in the Valencia Region. The yield (weight and number of fruits), the fruit setting, the water status of the soil and the trees, and the water productivity were analyzed in this paper.

## 2. Results and Discussion

Soil analyses show that the soil on the experimental site is fertile and has no health problems. Irrigation water also does not present any health problems in general or in the pomegranate crop. Regarding salinity, a ‘moderately tolerant’ crop such as pomegranate [21] should not have problems with irrigation water with an EC = 1.65 dS m^−1^. Indeed, the value of the EC of the saturated-soil extract (Ke = 2.48 dS m^−1^) is lower than the value of the salinity threshold (5 dS m^−1^ for pomegranate [22]), which is the parameter A (salinity from which the decrease in yield begins) of the Maas and Hoffman equation Yr = 100 − B (Ke − A), where Yr is the relative yield for any given soil salinity exceeding the threshold [23].

### 2.1. Growth Stages and Climatology

The growing season duration (considered from the first leaves sprouting to harvest) was 252 d in both 2022 and 2023; the duration, reference evapotranspiration (ETo), and crop coefficient (Kc) of each stage are presented in Table 1.

**Figure 1:**
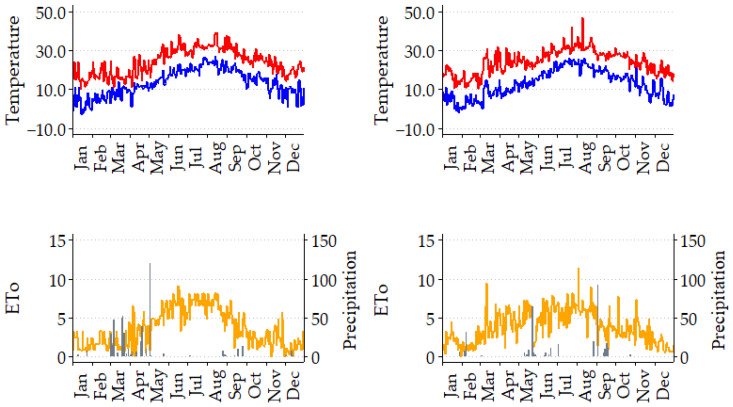
Seasonal variation of daily maximum (Tmax; red) and minimum (Tmin; blue) temperatures (°C), reference evapotranspiration (ETo; mm; yellow), and precipitation (mm; gray vertical bars) in 2022 (**left**) and 2023 (**right**).

Figure 1 shows the most significant climatological data for both experimental seasons, maximum and minimum temperature, ETo, and precipitation. Although the difference between the total ETo values throughout the season was not very large, 5.4% (1110 mm in 2022 and 1169 mm in 2023), this difference increases up to 75% when considering the ETo recorded during April and May (147 mm in 2022 and 258 mm in 2023). It should be noted that while 2022 behaved like an average year in terms of rainfall (505 mm during the growing period), with significant rainfall recorded in spring, 2023 was very hot and dry (352 mm), with almost half of this precipitation recorded in September. The Pe recorded in the two seasons was different considered both throughout the season (364 mm in 2022 and 301 mm in 2023) and in seasonality. While the 2022 spring was very rainy (227 mm during March and April), the 2023 one was very dry (less than 2 mm). On the other hand, while in September 2022, only 24 mm was recorded, 107 mm was registered in 2023.

### 2.2. Soil and Plant Water Status

The differences in the climatology recorded in the two years and the growth of the trees throughout the experiment, with the consequent increase in Kc (Table 1), led to an increase in the IWA over the years of study. In 2022, the IWA was 316, 284, 276, and 163 mm in Control, RDI1, RDI2, and SDI, respectively, while in 2023, it was 463, 426, 430, and 237 mm. SDI meant a water saving of approximately 50% compared to the Control, as expected, indicating that the objective of providing 50% of the Control was met. In RDI1, water savings of 11% in 2022 and 8% in 2023 were achieved compared to Control, while in RDI2, the savings were 13% and 7% in 2022 and 2023, respectively.

**Figure 2:**
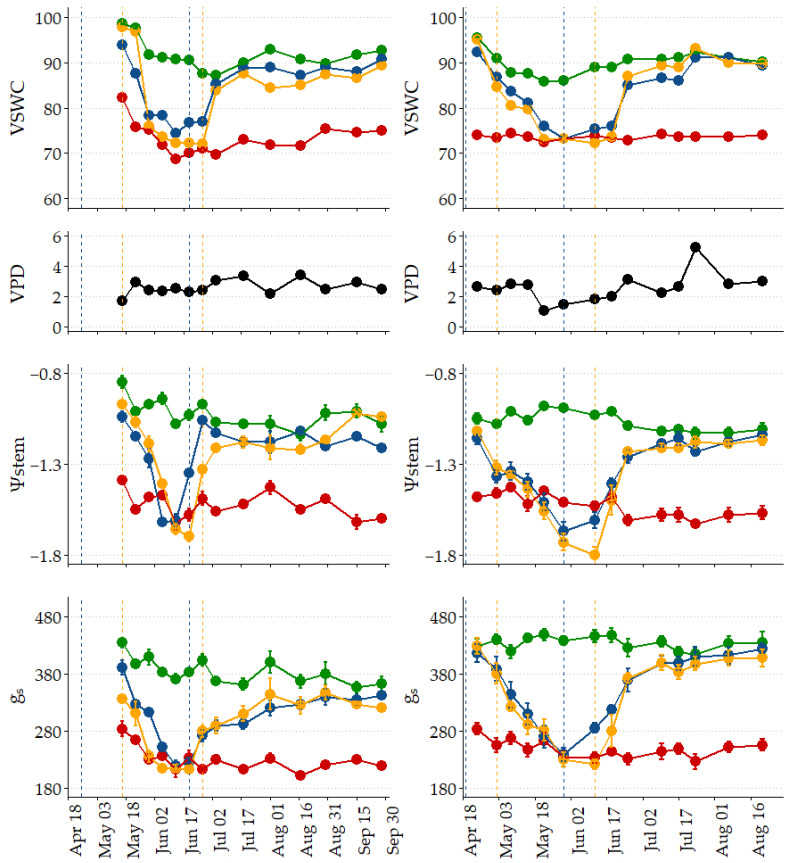
Seasonal variation of volumetric soil water content (VSWC), air vapor pressure deficit (VPD in black), stem water potential (Ψstem), and stomatal conductance (g_s_) registered in the different irrigation treatments (Control in green; RDI1 in blue; RDI2 in yellow; SDI in red) in 2022 (**left**) and 2023 (**right**). All measures correspond to midday. Discontinuous vertical lines represent the start and end of the water restriction periods in RDI1 (blue) and RDI2 (yellow). Vertical bars represent the standard error.

Figure 2 shows the seasonal variation of midday volumetric soil water content (VSWC), air vapor pressure deficit (VPD), stem water potential (Ψstem), and stomatal conductance (g_s_). The values presented correspond to the midday, when stem water potential and stomatal conductance were measured before the corresponding irrigation event.

In general terms, it can be stated that the objective of starting irrigation when the VSWC value reached 90% of the field capacity was met. The values of VSWC, Ψstem, and g_s_ recorded in SDI (73.7%, −1.56 MPa, 226 mmol m^−2^ s^−1^, on average before the evaluated irrigation events, respectively) were markedly lower than those corresponding to the Control strategy (91.9%, −1.04 MPa, 387 mmol m^−2^ s^−1^, respectively). The values corresponding to RDI were close to those of SDI during the water restriction phases and to those of the Control during the rest of the crop cycle. The VPD values ranged between 1.30 and 5.19 kPa, obtaining in 2022 a maximum value of 3.4 kPa (on 17 August) and in 2023 of 5.19 kPa (on 24 July).

Ψstem values at midday decreased as the season progressed (Figure 2), in agreement with what has been reported in the literature for ‘ME’ pomegranate [11,14,24]. In RDI strategies, a rapid recovery of Ψstem values was recorded when full irrigation was resumed, as also observed by Mellisho et al. (2012) and Martínez-Nicolás et al. (2019). The midday Ψstem values recorded in similar research in ‘ME’ for Control plants ranged between −0.6 and −1.02 MPa, while those in RDI plants in the restriction’s periods ranged between −1.61 and −2.18 MPa [11,14,24,25]. Parvizi et al. [26] obtained lower values (−2.6 and −3.3 MPa for Control and SDI at 50% ETc, respectively) with ‘Rabat’ pomegranate in Iran. The minimum value of midday Ψstem recorded in SDI is like the minimum obtained by [11] in the Control treatment; therefore, it does not seem that there was significant water stress in any of the strategies tested. 

Regarding g_s_, there was also a general trend to decrease as the season progressed (Figure 2), oscillating from 435 to 356 mmol m^−2^ s^−1^ for the Control plants and from 276 to 202 mmol m^−2^ s^−1^ for the SDI plants. RDI plants behaved similarly to SDI during the respective water restriction periods and like Control plants during the initial and final phases. These values agree with those obtained by [14,24], but they are higher than those presented by [17] (from 220 to 50 mmol m^−2^ s^−1^ for Control and from 230 to 30 mmol m^−2^ s^−1^ for SDI) and are much higher than those presented by [26] (120 to 20 mmol m^−2^ s^−1^).

**Figure 3:**
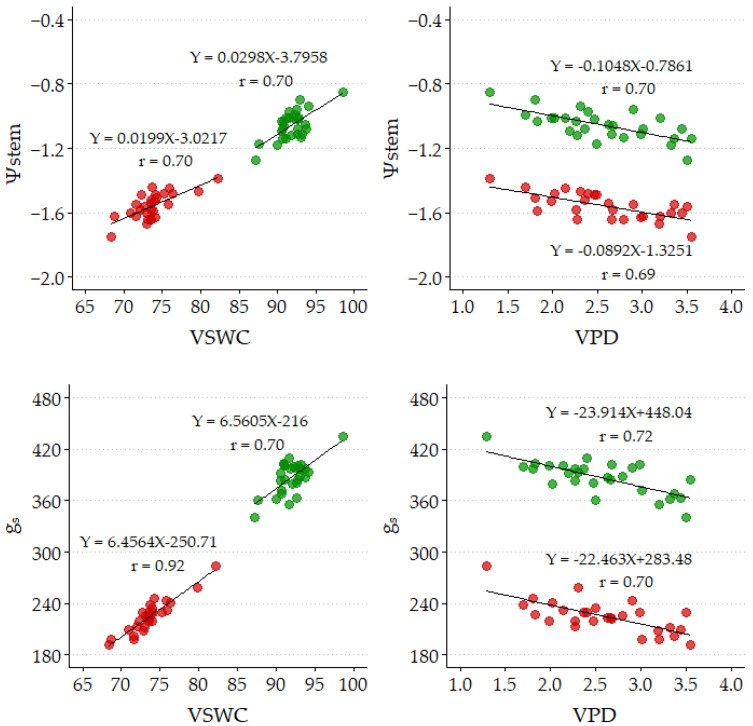
Linear correlation between midday stem water potential (Ψstem, MPa) and stomatal conductance (g_s_, mmol m^−2^ s^−1^) with volumetric soil water content [VSWC, (%); **left**] and air vapor pressure deficit (VPD, kPa; **right**) for Control (green) and sustained deficit irrigation (SDI; red) strategies. Obtained from values corresponding to 2022 and 2023.

**Figure 4:**
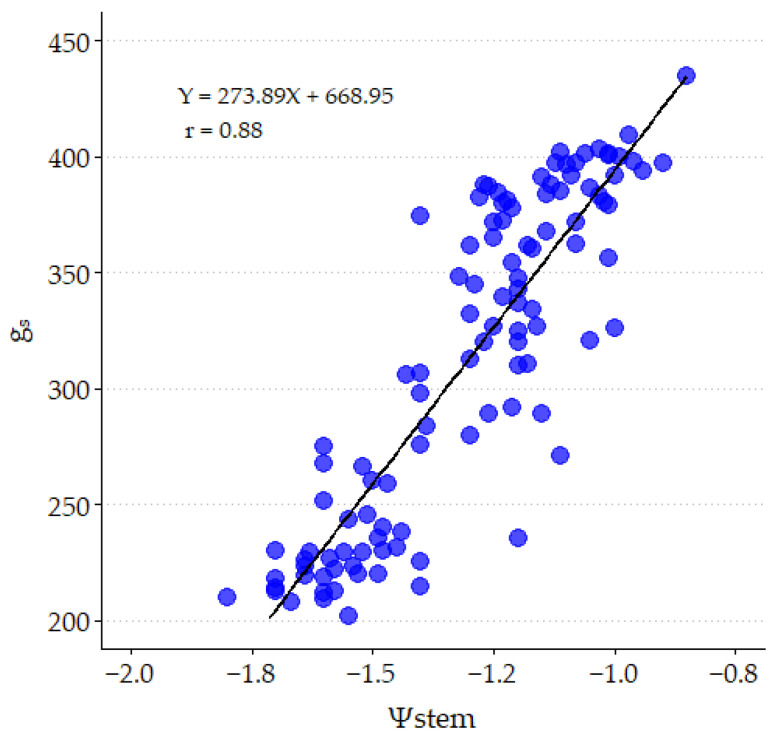
Linear correlation between midday stomatal conductance (g_s_, mmol m^−2^ s^−1^) and stem water potential (Ψstem, MPa), obtained from values corresponding to 2022 and 2023.

Significant relationships (*p* ≤ 0.05) have been found between Ψstem and g_s_ with VSWC (positive) and VPD (negative) for Control and SDI strategies. Figure 3 presents these relationships adjusted with the 2022–2023 data, whose correlation coefficients (≥0.69) are similar to those obtained by [11] with ‘ME’. These relationships for RDI presented lower correlation coefficients since they considered both measurements carried out with water restrictions and full irrigation. A positive linear relationship is observed between g_s_ and Ψstem, with a high correlation coefficient (0.88; Figure 4).

With the strategies tested, decreases in Ψstem and g_s_ are observed when VSWC decreases (Figure 3), so it seems that ‘ME49’ trees have effective control of the plant water status by reducing transpiration due to stomatal closure, as reported by [27], while maintaining Ψstem in narrow limits during the hydric restrictions in RDI strategies and throughout the crop cycle in the SDI strategy. That is, pomegranate trees ‘ME49’ presented a typical anisohydric behaviour as reported by [28]. Rodríguez et al. [15] reported that pomegranate plants confront water stress by developing stress avoidance and tolerance mechanisms, which are complementary and take place gradually. When water is restricted, leaf conductance decreases to control water loss via transpiration and avoid leaf turgor loss (stress avoidance mechanism). When the deficit irrigation is more severe and maximum stress levels are developed, active osmotic adjustment is triggered, contributing to the maintenance of leaf turgor (stress tolerance mechanism). According to these authors, only stress avoidance mechanisms would have occurred in the present study since only a mild deficit was obtained.

### 2.3. Tree Performance

Table 2 presents the size of the trees at the end of the 2023 season, expressed in terms of canopy height and diameter and trunk perimeter, as well as growth, expressed by the increase in the values of these parameters throughout the experiment (end 2021–end 2023). The final size of the trees was larger than that of 2021 (height: 286 cm; diameter: 258 cm; trunk perimeter: 33.6 cm; mean values corresponding to the Control trees). RDI1 led to a lower height and trunk perimeter than RDI2, which would reduce the canopy size in the long term, which could result in lower yields. SDI reduced the canopy’s height and diameter and the trunk perimeter growth compared to Control and RDI2 (and the canopy diameter compared to RDI1). These results are similar to those obtained by [11] in ‘ME’ and to those obtained in ‘Wonderful’ by [29] in Chile and [30] in Italy. Since these values also depend on the values corresponding to the initial situation, the relative growth was analyzed, observing that the trees corresponding to the SDI strategy presented a lower relative growth than the trees corresponding to the other irrigation strategies (with statistical significance in the relative growth of the trunk perimeter). The high values of the percentage of the residual sum of squares (up to 88.4% for canopy diameter relative growth; Table 2) indicate an important variability between replicates, as reported by [31] in ‘Wonderful’ pomegranate in California. These authors reported that water deficit reduced the tree canopy size, although due to the notable variability between replications, the effect of irrigation strategies on canopy growth was not statistically significant.

The total number of flowers was affected (*p* ≤ 0.01; Table 3) by the growing season and the irrigation strategy but not by their interaction, so that in 2023, many more flowers were produced than in 2022 (more than tripled), highlighting the high number of flowers obtained in the Control strategy.

Flowering is induced by endogenous and exogenous signals that initiate parallel pathways [32]. Exogenous signals include temperature, photoperiod, and stress, while endogenous signals include plant age, fruit load, and nutritional and hormonal status. In the present experiment, regarding external signals, the photoperiod was similar in the two seasons. Table 4 shows the chill accumulation calculated for each season using the three main and most used models [33]—hours under 7.2 °C [34], Utah [35], and Dynamic [36] —and heat accumulation calculated using the Anderson model [37].

The most used terminology for the dormancy of fruit trees in temperate zones [38] is that proposed by Lang et al. in 1987 [39], who defined dormancy as the temporary suspension of visible growth of any plant structure containing a meristem. They distinguished between para-, endo-, and eco-dormancy phases. Para-dormancy refers to growth suppression imposed on organs by other tree structures (e.g., apical dominance) due to inhibitory molecules’ production and/or action. During the endo-dormancy phase, growth is impossible even under suitable temperature conditions, as buds during winter rest must be exposed to a low-temperature phase (chill needs). Once endo-dormancy has passed, they must be exposed to warm temperatures during the eco-dormancy phase (heat needs) to flower. In 2023, there was less chill accumulation and greater heat accumulation than in 2022. The differences in chill accumulation recorded in this experiment (with higher values in 2022) do not justify the differences in flowering intensity. However, the approximately 4% higher heat accumulation recorded in 2023 compared to 2022 is probably the cause of the earlier sprouting of both vegetative and reproductive buds. The recorded chill and heat accumulation values are in line with those obtained by [40] in ‘ME’ and confirm that ‘ME49’ is much less demanding in chill accumulation than other cultivars/accessions, such as those grown in Iran [41,42]. Regarding heat accumulation, the GDH registered was higher than reported in Iran [41] and Himachal Pradesh (India) [43].

The last exogenous signal reported by [32] is the fact that plants are under some kind of stress; no stress was recorded during bud dormancy nor after it, with the only difference being in the irrigation treatment; in this sense, the Control strategy led to a greater number of hermaphrodite flowers than the RDI1 and SDI strategies. Although despite water restriction, the trees did not suffer water stress (as mentioned above), the results obtained are consistent with those obtained by [42] in the sense that with the same chill accumulation, the greatest flowering was obtained with the Control strategy, that is, without any water restriction.

Regarding endogenous signals, during the experiment, the trees grew in height (from 13 to 21%) and diameter (from 16 to 24%), which would have increased their flowering capacity. Our result agrees with that reported by [44], who stated the possibility that SDI and RDI decreased growth sprouting and, consequently, the number of flowers formed on these shoots. On the other hand, the yield obtained by the Control trees in 2021 (27 kg tree^−1^) was much lower than that obtained in 2022 (58 kg tree^−1^), which in turn was much lower than in 2023 (83 kg tree^−1^). It seems that the fruit load of the trees in each season should have been independent of the flowering of the next, not showing biennial (or alternate) bearing. Regarding the nutritional status of the trees, all of them received identical fertilization, and the nutrient content recorded in the foliar analyses had value ranges at levels considered normal [45,46], with no significant differences detected between the values corresponding to the different irrigation strategies. Table 5 shows the average foliar content (for the two seasons) of the nutrients analyzed.

About fruit setting (Table 3), it is observed that in 2023 many more fruits set than in 2022 and that RDI1 and RDI2 led to a higher percentage of fruit set (*p* ≤ 0.01) than Control and SDI. The interaction analysis (*p* ≤ 0.01) shows that the lowest value in 2022 corresponded to SDI, while in 2023, it corresponded to Control. According to [47], with the production of a high number of flowers, there is high competition between flowers for synthesized carbohydrates, which would justify the lowest percentage of fruit set in 2023 in the Control strategy. As expected, due to the high number of hermaphrodite flowers produced, and despite the high percentage of fallen reproductive organs (100—set fruits), the percentage of thinned fruits about the total flowers in 2023 was higher (*p* ≤ 0.01) than in 2022, highlighting RDI1, which recorded the lowest value in 2022 (*p* ≤ 0.01) and the highest in 2023.

The percentage of fruits harvested compared to the total number of hermaphrodite flowers was greater in 2022 than in 2023 (*p* ≤ 0.01; Table 3), and the two RDI strategies led to higher percentages (*p* ≤ 0.01) than the Control and SDI; from the analysis of the interaction (*p* ≤ 0.05), it is observed that the lowest value in 2022 corresponded to SDI, while in 2023 it corresponded to the Control. In young-bearing pomegranate trees, there appears to be a balance between the water available to the plant and the competition for assimilates between yield and vegetative growth [20] as well as between different fruits [47]. In this sense, the Control strategy led to the highest tree growth and the highest number of flowers, and therefore to the greatest competition, both between shoots and fruits and between fruits, and consequently to the highest percentage of fallen flowers and fruitlets in 2023. The balance between the water available and the competition for assimilation between flowers and fruitlets led to the lowest percentage of fruits harvested compared to the total number of hermaphrodite flowers in SDI in 2022. This result is consistent with [14], who stated that water stress decreased shoot growth but did not affect the number of viable flowers.

### 2.4. Total and Marketable Yield, Water Productivity, and Yield Response Factor

Both irrigation strategies and growing season affected total and marketable yields (Table 6), resulting in significant interactions between both factors for yield in terms of weight (*p* ≤ 0.05 for total and *p* ≤ 0.01 for marketable yield). The total yield (kg tree^−1^) obtained in 2023 was higher than that obtained in 2022 (on average 43%), and in both seasons, SDI led to the lowest value. As for the number of fruits, the production in 2023 was, on average, 66% greater than in 2022, with the lowest value corresponding to SDI.

In terms of marketable yield, the average value obtained in 2023 exceeded that obtained in 2022 (Table 6), both in weight (by 40%) and in number (by 68%). The high average marketable yield obtained in 2023 (51.3 kg tree^−1^) exceeds that obtained by [14] with ‘ME’ in 2016 (40.6 kg tree^−1^), while that obtained in 2022 (36.8 kg tree^−1^) slightly exceeds that obtained by these authors in 2015 (35.7 kg tree^−1^). The number of marketable fruits obtained per tree in 2023 (on average 137) was much higher than in 2022 (on average 82), being higher and lower, respectively, than those obtained by [14] (90–99 in the different growing seasons). RDI1 led to the highest marketable yield (not significantly differing from the Control or RDI2), coinciding with the results obtained by Intrigliolo et al. (2013), as well as those obtained by [14], which found no negative effect of the deficit irrigation. The SDI strategy led to the lowest marketable yield (*p* ≤ 0.01) both in weight and number. It should be noted that due to the severe incidence of cracking recorded in SDI in 2023, the number of marketable fruits obtained in SDI was only 8% higher than in 2022, while in the rest of the irrigation strategies, the increase was, on average, 80%. Regarding the unit weight, the analysis of the growing season–irrigation strategy interaction (*p* ≤ 0.01) shows that the fruits obtained in 2022 were larger (higher unit weight) than those of 2023, which would be related to the higher number of marketable fruits obtained that year. On the other hand, in 2023, SDI led to the lowest value, probably due to the high incidence of cracking, which would affect the largest fruits, leaving the smaller fruits, and therefore those with lower unit weight as commercial fruits.

Regarding the WP, from the analysis of the season–irrigation strategy interaction (*p* ≤ 0.01), it is remarkable that SDI led to the highest absolute value (11.5 kg m^−3^) in 2022 and the lowest (7.4 kg m^−3^) in 2023. These different values are mainly due to the higher IWA in 2023 compared to in 2022 (163 mm in 2022 and 237 mm in 2023) and to a lesser extent to the lower marketable yield obtained in 2023 (18.75 t ha^−1^ in 2022 and 17.43 t ha^−1^ in 2023), a consequence in turn of the different flowering and fruit setting and, above all, the high incidence of cracking in 2023. In the two growing seasons, a lower value was obtained with the control strategy (7.3 and 7.7 kg m^−3^ in 2022 and 2023, respectively) than with the RDI strategies.

Regarding Ky, values greater than 1 indicate that the crop’s response is very sensitive to water deficit. In contrast, a Ky value lower than 1 means the crop is more tolerant to water deficit. When Ky is equal to 1, the reduction in yield is directly proportional to the reduction in water use. RDI strategies led to higher marketable yields than Control, so Ky would be negative; thus, it was only determined in SDI. In 2022, with a climate that can be considered normal, Ky was 0.38, meaning that ‘ME 49’ behaved as tolerant, but instead, in 2023, with a very hot summer, in which a high incidence of cracking was added to the lower performance usually obtained with SDI, Ky was 1.05, meaning that it behaved as sensitive, with a reduction in yield almost directly proportional to the reduction in water use.

### 2.5. Considerations for the Future

Despite the interesting works published on the pomegranate tree, there are still aspects that should be studied in further depth in the future, such as the use of multispectral cameras installed or not in drones, which would allow researchers to accurately analyze the growth of the trees as a response to the water deficit and also estimate parameters such as leafiness and/or leaf area index.

Given that the percentage of hermaphrodite flowers is proportional to the plant’s bearing ability and determines the degree of fruit set [48] and that not all bibliographic references agree on the influence of RDI on the hermaphrodite/male ratio [44], the aim is to continue this study by analyzing the influence of deficit irrigation (both RDI and SDI) on the number and proportion of hermaphrodite flowers and their setting, as well as its influence on yield. Future studies will analyze the exogenous (cold and water stress) and endogenous (particularly hormonal) pathways that control flowering and their effect on changes in gene expression and protein and metabolite levels in pomegranate leaves and shoots. Regulated deficit irrigation with more severe water restrictions would allow us to study, on the one hand, the possible active osmotic adjustment, which would favor the maintenance of leaf turgor (stress tolerance mechanism) and, on the other hand, the corresponding productive response and the water productivity. Sustained deficit irrigation with fewer restrictions is probably also an appropriate option in situations of significant water scarcity that should be addressed.

## 3. Materials and Methods

### 3.1. Experimental Site Description

The field studies were conducted at the Cajamar Experimental Centre in Paiporta, Valencia, Spain (39.4175 N, 0.4184 W), over two consecutive growing seasons (2022 and 2023). The soil at the site is deep with a silt loam texture; according to the USDA Soil Taxonomy, it is classified as Petrocalcic Calcixerepts [49]. At the beginning of the experiment, the soil was very slightly alkaline (pH = 8.17), slightly saline [EC (1:2.5) = 3.03 dS m^−1^], and highly fertile [on average: organic matter = 1.4%; phosphorous = 171.4 mg kg^−1^ (Olsen); potassium = 581 mg kg^−1^ (ammonium acetate extract)]. The soil was uniform in the root zone depth (50 cm), and volumetric soil water contents (VSWC) at the field capacity and permanent wilting point were (on average) 28.7% and 16.1%, respectively, for 2022 and 28.8% and 16.1% for 2023. Irrigation water was pumped from a well, which averaged EC 1.65 dS m^−1^, pH 7.58, and 23.7 mg kg^−1^ N-NO_3_- content. According to Papadakis’s agroclimatic classification [50], the climate is subtropical Mediterranean (Su, Me) with hot and dry summers. The annual average rainfall is approximately 450 mm, irregularly distributed throughout the year, mostly in autumn and the beginning of spring.

### 3.2. Plant Material and Agronomic Details

The experimental orchard was established in 2015 from vegetatively propagated plants of ME49, a selected clone of ‘Mollar de Elche’. This clone is one of the most appreciated and used in the area due to its adequate adaptation to edaphoclimatic conditions, high productivity, and great acceptance. Pomegranate tree spacing followed a 3.5 m × 4.5 m pattern, and trees were trained to the vase-shaped system.

The agricultural practices, including covering the soil with a perennial grass (Arbovert Perenne; 100 kg ha^−1^; Intersemillas, València, Spain), fertilization, pruning, and fruit thinning, were those commonly used by farmers in the area. Specifically, on 17 and 30 June 2022 and 6 and 26 June 2023, a light manual thinning was carried out to eliminate multiple fruits, leaving one–two fruits per node, and to promote an increase in fruit size at harvest by reducing competition between fruits by eliminating late-flowering fruits. The incorporation of nutrients (150–75–180 kg ha^−1^ year^−1^ N–P_2_O_5_–K_2_O) was carried out through fertigation, integrating the criteria indicated by [14,51].

### 3.3. Irrigation Strategies

In all four strategies, irrigation started with the sprouting of the first leaves (BBCH 10; 25 February 2022 and 22 February 2023) and finished with the second harvest (BBCH 81; 3 November 2022 and 31 October 2023).

The irrigation in the Control strategy was performed according to the criteria commonly used by the growers in the area [15], consisting of replacing the crop water needs [11,14] and at the same time allowing soil moisture to reach a depth of 50 cm, without any water loss being recorded at depth. Trees subjected to SDI received 50% of the water applied to Control trees in each irrigation event. RDI trees were irrigated with 33.3% of the crop water needs during the corresponding restriction period (Table 7) and with 100% outside this period. Differential irrigation started at the beginning of the 2021 season to not interfere with the establishment of trees. Table 8 presents the percentage of irrigation water requirements applied during the irrigation season in each irrigation strategy.

The irrigation water requirement (IWR) for each irrigation event was determined with the following equation: IWR = (ETc-Pe)/Ef, where ETc (mm) is the crop evapotranspiration, Pe is the effective precipitation (mm), determined from rainfall data using the US Bureau of Reclamation method [52], and Ef is the irrigation efficiency. This Ef was 0.97, considering the distribution uniformity (0.97; in situ determined) and negligible leaching requirement [EC of the irrigation water 1.65 dS m^−1^; EC of saturated-soil extract = 2.48 dS m^−1^]. The irrigation frequency ranged between daily when the water requirements were maximum (summer) and every three days when the water requirements were minimum (spring). Irrigation dose was determined retrospectively to replace the water requirements of the previous period.

Following the criteria described by [53], ETc was determined by equation ETc = ETo × Kc, using the reference evapotranspiration (ETo) and the single crop coefficient (Kc), with values ranging from 0 to 0.72, which were proposed for local conditions by the *Instituto Valenciano de Investigaciones Agrarias* (IVIA) [54], adapting the duration of each stage to the growing cycle. Daily ETo (mm day^−1^) was determined using the FAO-56 Penman–Monteith equation (Equation (1); [53]):

(1) $${ET}_{0}=\frac{0.408∆\left(R_{n}-G\right)+\gamma \frac{900}{T+273}u_{2}\left(e_{s}-e_{a}\right)}{∆+\gamma \left(1+0.34u_{2}\right)}$$

where Δ is the slope of the saturation vapor pressure–temperature relationship at mean air temperature (kPa °C^−1^), R_n_ is the net radiation at the crop surface (MJ m^−2^ d^−1^), G is the soil heat flux (MJ m^−2^ d^−1^), T is the average air temperature (°C), u_2_ is the wind speed at 2 m height (m s^−1^), (es-ea) is the vapor pressure deficit (kPa), and γ is the psychrometric constant (kPa °C^−1^).

Plants were irrigated by a drip irrigation system with two lateral lines (Netafim Standard Irrigation PE Pipes 16/4, Netafim, Valencia, Spain) with six self-compensating emitters per tree (Netafim PCJ, 4.0 L h^−1^) spaced 0.50 m apart. In each strategy, the irrigation water applied was recorded by a water flow meter (MJ-LFC, Dn 22, NWM, Ningbo Water Meter Co., Ltd., Ningbo, China).

### 3.4. Volumetric Soil Water Content

Volumetric soil water content (VSWC; m^3^ m^−3^) was continuously monitored by ECH_2_O EC-5 capacitance sensors connected to an Em50 data logger using the ECH_2_O Utility software (Decagon Devices, Inc., Pullman, WA, USA). Factory calibration provides ± 3% accuracy for mineral soils and was, therefore, used directly. Two sensors per replicate were placed below an emitter at a 0.25 and 0.5 m depth, respectively. In previous experiments performed at the same experimental plot, it was stated that the maximum root density of pomegranate trees occurred at a depth of 0.25 m. The sensor located at 0.50 m depth made it possible to verify that the humidity reached this depth and that deep percolation was controlled. VSWC was measured and stored every 15 min, and its variation was used to determine the in situ field capacity. The irrigation event for each IS began when the VSWC in the Control dropped to 90% of the field capacity, applying the corresponding irrigation dose to each IS. This criterion was already satisfactorily used in preliminary studies. VSWC data were managed and presented as a percentage of field capacity, reducing the importance of sensor calibration.

### 3.5. Plant Water Status

Midday (12 h solar time) stem water potential (Ψstem) was assessed with a pressure chamber (Soil Moisture Equip. Corp. mod. 5100A, Santa Barbara, CA, USA), following the procedure described by Intrigliolo et al. (2013). Mature (fully expanded) leaves from the north-facing side and middle third of the tree were covered with silver foil and enclosed in plastic bags two hours before measurements. Under the same conditions, the midday leaf conductance (g_s_) was measured with a porometer (Delta T AP4, Delta-T Devices, Cambridge, UK) on the abaxial surface of the leaves. Both determinations were performed simultaneously in twelve leaves per treatment (two leaves per tree, two trees per block, and three replication blocks). In each growing season, eight measurements were taken before the corresponding irrigation event.

### 3.6. Tree Performance Determinations

Tree size was estimated by measuring the canopy height and diameter at three locations in four trees per replicate at the end of each growing season (after leaf fall). Similarly, the trunk perimeter was measured at the beginning of the experiment and the end of each growing season at 0.2 m above the ground. Relative growth in canopy height and in diameter, as well as trunk perimeter, was calculated as the ratio between the increment of each parameter during the experiment and their initial values.

The fallen reproductive organs (flowers and fruitlets) per tree were collected and recorded weekly and considered together with the number of set fruits to determine the number of hermaphrodite flowers. The number of set fruits was, in turn, defined as the number of fruits thinned and harvested.

Sampling to analyze the leaf nutrient concentrations was carried out on July 15 of each year, following the recommendations of experts [45,46]. Eighty fully expanded leaves per treatment were collected 20 leaves per tree (five leaves from each orientation: north, south, east, and west) at 1.5 m above ground level) from branches without developing fruits [55] and from 4 trees per replication block. The leaf analyses were carried out in the ‘Las Palmerillas’ laboratory in Cajamar, following the official analysis methods [56].

### 3.7. Chill and Heat Accumulation

Chill accumulation was calculated for each season from 1 November until 28 February, as reported by [40], using the three most used models [33] (hours under 7.2 °C [34], Utah [35], and Dynamic [36]). Heat accumulation was calculated as growing degree hours (GDH) for each season, from 1 January to 8 April [40], considering a base temperature of 4 °C, an optimum temperature of 25 °C, and a critical temperature of 36 °C (the temperature above which no appreciable growth occurs [37]).

### 3.8. Yield, Water Productivity, and Yield Response Factor

When fruit commercial maturity was reached (unit weight > 220 g [57] and total soluble solids > 14° Brix [14]), pomegranate fruits were manually harvested in two commercial pickings carried out on 13 October and 3 November 2022 and 16 and 31 October 2023. The total and marketable yield of fruits destined for the fresh market was determined, both in weight and in number, visually evaluating rejection due to alterations in the fruits (cracking, sunburn, small size, and other alterations, which mainly included scratches and deformed fruits).

Water productivity was calculated in physical terms as the relationship between marketable yield and the IWA, according to [5]. The yield response to water deficits during each growing season was determined according to [58] (1-Ya/Ym) = Ky (1 − ETa/ETm), where Ky is the yield response factor; Ya and Ym are the actual and maximum marketable yield (kg m^−2^), respectively; and ETa and ETm are the actual and maximum ETc (mm), respectively, being in turn calculated from soil water balance, considering negligible both the drainage and the variation in the volumetric soil water content [4].

### 3.9. Experimental Design and Statistical Analysis

The experiment was laid out in a randomized complete block design with three replicates. Each experimental plot was composed of three rows of trees with six trees each, of which the four central trees of the central row were analyzed; these trees were very similar in appearance (height, ground shaded area, and trunk cross-sectional), as [18] reported.

The results for the different parameters were evaluated by analysis of variance (ANOVA) using Statgraphics Centurion 19 [59]. Percentage data were arcsin transformed before analysis. Least significant difference (LSD) at a 0.05 probability level was used as the mean separation test. Initially, the data from each growing season were analyzed separately, with the result that in no case was the repetition block significant, so they were analyzed together without considering the repetition block to avoid third-order interactions.

## 4. Conclusions

With the strategies tested, a decrease in stem water potential (Ψstem) and leaf conductance (g_s_) is observed when soil moisture decreases, so ‘ME49’ pomegranate trees have effective control of the plant water status by reducing transpiration due to stomatal closure, maintaining the stem water potential in narrow limits during the hydric restrictions. With the regulated deficit irrigation strategies tested, a small water saving is achieved, of the order of 10% of the irrigation water requirement, without reducing the marketable yield or the unit weight of the fruits. Specifically, when the reduction is applied in the flowering–fruit set stage, a slight increase in marketable yield is obtained; this irrigation strategy leads to a slight reduction in the growth of both the height and the perimeter of the tree trunk. With the tested sustained deficit irrigation, savings equivalent to 50% of the irrigation water requirement are obtained but at the cost of a slight reduction in the growth of height and perimeter of the trunk and a significant reduction in commercial yield. The magnitude of this reduction depends on the climate of the growing season, so with very hot and dry summers, the reduction in commercial yield can reach 50%. On average, regulated and sustained deficit irrigation increases water productivity. Regulated deficit irrigation is an advisable irrigation strategy for pomegranate cultivation regardless of whether or not there is water restriction, while the tested sustained deficit irrigation would only be advisable in situations of significant water scarcity.

## Figures and Tables

**Table 1:** Duration, reference evapotranspiration (ETo), crop coefficient (Kc) values and irrigation water applied (IWA) in the different irrigation stages during 2022 and 2023.

Strategy	Period	2022				2023			
		Duration (d)	ETo (mm)	Kc	IWA (mm)	Duration (d)	ETo (mm)	Kc	IWA (mm)
Control									
	Entire cycle	252	1110.2	0.30–0.68	316.0	252	1169.3	0.30–0.72	463.4
SDI									
	Entire cycle	252	1110.2	0.30–0.68	163.4	252	1169.3	0.30–0.72	236.6
RDI1									
	Full irrigation	60	140.2	0.30–0.39	0.0	56	223.1	0.30–0.41	22.2
	Restriction	55	291.6	0.48–0.58	16.1	47	207.3	0.51–0.62	20.9
	Full irrigation	137	678.3	0.39–0.68	268.1	149	738.9	0.41–0.72	383.4
RDI2									
	Full irrigation	80	212.5	0.30–0.39	3.3	66	264.8	0.30–0.41	32.9
	Restriction	42	258.5	0.48–0.58	19.9	44	203.1	0.51–0.62	21.8
	Full irrigation	130	639.2	0.39–0.68	252.9	142	701.4	0.41–0.72	375.7

**Table 2:** Effect of irrigation strategy on both size [canopy height (H) and diameter (D) and trunk perimeter (TP)] and the corresponding relative growth for the entire experiment.

		Size (cm)						Relative Growth (%)					
		H		D		TP		H		D		TP	
Irrigation strategy (IS)													
	Control	344	ab	319.4	a	37.13	a	20.65		24.17		10.58	a
	RDI1	324	bc	318.0	a	35.04	b	18.45		23.01		8.22	a
	RDI2	352	a	333.1	a	37.06	a	21.56		27.57		8.46	a
	SDI	319	c	287.0	b	34.33	b	12.60		15.68		5.04	b
Sources (df)		% Sum of squares											
IS (3)		19.79	*	23.86	**	25.90	**	15.19	ns	11.57	ns	22.40	*
Residual (44)		80.21		76.14		74.10		84.81		88.43		77.60	
Standard deviation		28.86		31.51		2.17		8.61		12.52		3.85	

Mean values followed by different lowercase letters in each column indicate significant differences at *p* ≤ 0.05 using the LSD test. df: degrees of freedom; ns: no significant difference; ** (*): significant differences at *p* ≤ 0.01 (*p* ≤ 0.05).

**Table 3:** Effect of growing season and irrigation strategy on the number of hermaphrodite flowers per tree and percentage of set, thinned, and harvested fruits in relation to the hermaphrodite flowers.

		Hermaphrodite Flowers (No Tree^−1^)		Set Fruits (%)		Thinned Fruits (%)		Harvested Fruits (%)	
Growing season (GS)									
	2022	219.15	b	78.12	b	8.03	b	70.10	a
	2023	723.23	a	45.90	a	9.92	a	36.01	b
Irrigation strategy (IS)									
	Control	537.46	a	59.60	b	9.82		49.78	b
	RDI1	436.63	b	65.71	a	8.75		56.96	a
	RDI2	476.33	ab	63.49	a	8.88		54.61	a
	SDI	434.33	b	59.25	b	8.44		50.87	b
GS × IS									
	2022—Control	239.50		79.39	a	10.00	ab	69.39	bc
	2022—RDI1	223.50		80.03	a	6.16	c	73.87	a
	2022—RDI2	211.17		79.54	a	8.51	bc	71.03	ab
	2022—SDI	202.42		73.52	b	7.43	bc	66.09	c
	2023—Control	835.42		39.81	e	9.64	ab	30.17	f
	2023—RDI1	649.75		51.38	c	11.33	a	40.05	d
	2023—RDI2	741.50		47.43	cd	9.24	ab	38.19	de
	2023—SDI	666.25		44.99	d	9.45	ab	35.65	e
Sources (df)		% Sum of squares							
GS (1)		79.09	**	85.73	**	7.42	**	90.33	**
IS (3)		2.17	*	2.39	**	2.21	ns	2.58	**
GS × IS (3)		1.31	ns	1.69	**	8.90	*	0.80	*
Residual (88)		17.44		10.19		81.47		6.29	
Standard deviation		123.61		5.78		3.28		4.7	

Mean values followed by different lowercase letters in each column indicate significant differences at *p* ≤ 0.05 using the LSD test df: degrees of freedom; ns: no significant difference; ** (*): significant differences at *p* ≤ 0.01 (*p* ≤ 0.05).

**Table 4:** Chill accumulation calculated for each season using three models: hours under 7.2 °C, Utah, and Dynamic. Heat accumulation calculated using the Anderson model.

Growing Season	Chill Accumulation			Heat Accumulation
	Hours < 7.2 °C	Utah	Dynamic	Anderson
	(h)	(Chill Units)	(Chill Portions)	(GDH)
2021–2022	395	488.5	53.92	23,052.3
2022–2023	331	333.5	49.58	24,047.4

**Table 5:** Leaf macronutrient (nitrogen, phosphorus, potassium, calcium, and magnesium; %) and micronutrient (iron, copper, manganese, and zinc; mg L^−1^) concentrations corresponding to adult leaves (15 July) from ‘Mollar de Elche’ clone 49 pomegranate trees. Average values of four trees per block of repetition and the two growing seasons.

	Macronutrients					Micronutrients			
	N	P	K	Ca	Mg	Fe	Cu	Mn	Zn
Control	2.04	0.16	0.86	1.71	0.38	124.33	8.50	34.00	19.33
RDI1	1.88	0.15	0.88	1.73	0.35	131.33	6.50	33.33	17.67
RDI2	1.92	0.15	0.91	1.62	0.38	144.83	7.50	33.33	18.50
SDI	1.82	0.14	0.83	1.68	0.41	118.50	7.00	32.67	17.17

**Table 6:** Effect of growing season and irrigation strategy on the total yield, marketable yield (including unit weight: UW), and water productivity (WP, in terms of kg m^−3^).

		Total Yield				Marketable Yield						WP (kg m^−3^)	
		(kg Tree^−1^)		(No Tree^−1^)		(kg Tree^−1^)		(No Tree^−1^)		UW (g)			
Growing season (GS)													
	2022	57.86	b	153.58	b	36.75	b	81.88	b	448.89	a	9.29	a
	2023	82.74	a	255.46	a	51.25	a	137.00	a	372.59	b	8.26	b
Irrigation strategy (IS)													
	Control	73.01	a	206.96	ab	46.30	b	115.75	a	409.36		7.50	b
	RDI1	75.75	a	211.42	a	51.23	a	125.75	a	417.32		9.13	a
	RDI2	76.48	a	215.63	a	49.98	ab	124.58	a	416.67		9.06	a
	RDS	55.97	b	184.08	b	28.49	c	71.67	b	399.61		9.42	a
GS × IS													
	2022—Control	60.43	b	165.83		36.09	bc	83.33	b	431.51	b	7.25	e
	2022—RDI1	62.00	b	164.92		40.28	b	89.50	b	448.90	ab	9.00	bc
	2022—RDI2	60.16	b	150.42		41.09	b	89.25	b	465.56	a	9.45	b
	2022—RDS	48.87	c	133.17		29.52	cd	65.42	c	449.58	ab	11.48	a
	2023—Control	85.58	a	248.08		56.52	a	148.17	a	387.21	c	7.74	cde
	2023—RDI1	89.51	a	257.92		62.18	a	162.00	a	385.73	c	9.26	b
	2023—RDI2	92.81	a	280.83		58.87	a	159.92	a	367.79	cd	8.68	bcd
	2023—RDS	63.08	b	235.00		27.45	d	77.92	bc	349.64	d	7.37	de
Source (df))		% Sum of squares											
GS (1)		44.03	**	59.53	**	23.76	**	41.07	**	58.29	**	6.33	*
IS (3)		19.96	**	3.41	*	37.70	**	26.52	**	2.04	ns	13.46	**
GS × IS (3)		3.22	*	1.83	ns	10.59	**	8.29	**	5.55	**	20.14	**
Residual (88)		32.78		35.23		27.95		24.11		34.12		60.07	
Standard deviation		11.21		40.93		8.22		22.06		30.48		1.66	

Mean values followed by different lowercase letters in each column indicate significant differences at *p* ≤ 0.05 using the LSD test. df: degrees of freedom; ns: no significant difference; ** (*): significant differences at *p* ≤ 0.01 (*p* ≤ 0.05).

**Table 7:** Irrigation restriction periods in the regulated deficit irrigation strategies for 2022 and 2023.

Strategy	Beginning	End	2022		2023	
			From	To	From	To
RDI1	The end of stage 1 (BBCH 51)	Young fruit of second flowering (BBCH 71)	26 April	19 June	19 April	4 June
RDI2	First open flower (BBCH 61)	Fruit growth of second flowering (BBCH 73)	16 May	26 June	29 April	11 June

**Table 8:** Irrigation water applied corresponding to irrigation strategies (IS) assayed, expressed as a percentage of the irrigation water requirements applied.

IS	February	March	April			May	June			July	August	September	October
Control	100%												
RDI1	100%			33.3%				100%					
RDI2	100%				33.3%				100%				
SDI	50%												

## Data Availability

Dataset available on request from the authors.
